# Understanding the Effects of In-Service Temperature and Functional Fluid on the Ageing of Silicone Rubber

**DOI:** 10.3390/polym11030388

**Published:** 2019-02-26

**Authors:** Sima Kashi, Mandy De Souza, Salwan Al-Assafi, Russell Varley

**Affiliations:** 1 Institute for Frontier Materials, Deakin University, Waurn Ponds, VIC 3216, Australia; mandy.desouza@deakin.edu.au (M.D.S.); russell.varley@deakin.edu.au (R.V.); 2 Quickstep Technologies Pty Ltd, Waurn Ponds, VIC 3216, Australia; sal-assafi@quickstep.com.au

**Keywords:** temperature, silicone rubber, thermal properties, mechanical properties, ageing

## Abstract

With an organic/inorganic hybrid nature, silicone elastomers are amongst the most versatile engineering materials, exploited in a wide range of applications either as end-products or in manufacturing processes. In many industrial machines, silicone components are exposed to in-service conditions, such as high or low temperatures, contact with functional fluids, mechanical loading, and deformations, which can adversely affect these components and reduce their lifespan, leading to machine failure in turn. The present study investigates the behaviour of a silicone component of a manufacturing equipment and the variations in the part’s properties due to in-service conditions (temperature, exposure to heat transfer fluid, and mechanical deformation) to develop a monitoring tool. An experimental design was employed to study the main and the interaction effects of temperature (22 °C, 180 °C), medium (air, synthetic heat transfer fluid), and strain (0%, 200%) on the silicone component’s properties. Results showed that while the chemistry of the component remains intact, its thermal and in particular mechanical properties are largely influenced by the in-service conditions. Consequently, leading to a physical rather than a chemical failure of the component and limiting its service life. Statistical analysis revealed that high temperature and the exposure to the heat transfer fluid have the most sever effects. Moreover, these two manufacturing parameters were found to have a significant interaction with one another, whose effect cannot not be neglected.

## 1. Introduction

Having an inorganic/organic hybrid nature, silicone rubbers fall within a material regime between silicate minerals and organic polymers with attributes of both sides [1]. Silicone rubbers are known for their high elasticity, low surface energy, high hydrophobicity, wide working temperature, exceptional weatherability, significantly high electrical resistivity, and biocompatibility [2,3,4,5,6,7]. This unique combination of outstanding properties places silicone rubbers among the most versatile materials with applications in industries as different as aerospace and automotive, electronics, power transmission and distribution, household and leisure, food sector, building industry, health and medical, and pharmaceutical [7,8].

Nevertheless, despite excellent properties and durability, silicone rubbers are still prone to deterioration depending on their service environment, which can eventually lead to their failure. Therefore, it is important to understand their behaviour over time under the effect of the in-service conditions, i.e., high or low temperatures, mechanical load, or chemical attack. Due to the wide application of silicone rubbers as electrical insulators in electrical apparatus, power transmission, and distribution systems, etc. [9,10,11,12,13], the studies on the ageing behaviour of silicone rubbers have mostly focused on weatherability, UV radiation ageing, and dielectric breakdown [10,11,13,14,15]. In contrast, there have been few studies on the deterioration of silicone rubber properties in other applications. Recently, Bernardi et al. [16] investigated the cyclic deformation and fracture behaviour of silicone-rubber elastomers in soft biomedical implants. In another recent study, Wu et al. [17] examined the thermal oxidation ageing of silicone rubber with a focus on its application as a sealant for equipment. The ageing behaviour of silicone rubber sealants in fuel cell applications has also been investigated in another recent study [18]. With the increased usage of silicone rubbers in alternative applications, such as industrial machinery and processes, it has become crucial to understand their ageing behaviour and the effects of in-service conditions on properties to prevent failure. Many industrial machines have silicone rubber components, which are in continuous contact with various lubricants, heat transfer fluids while at either very high or low temperatures, or also during mechanical loading. These conditions have the potential to severely affect the rubber’s properties and shorten its useful service life.

The present article reports on the accelerated ageing study of a silicone rubber in different environments and ageing conditions selected to replicate the most extreme conditions experienced by a silicone rubber part in a specific industrial process, where the rubber is exposed to polyalkylene glycol as a heat transfer fluid at high temperature and large mechanical deformations. The aim of the study was to investigate the variations in the properties of the silicone rubber due to ageing with a focus on mechanical and thermal properties. In our previous work [2], we monitored the properties of silicone rubber over a six-week period for one ageing environment, where the rubber samples were submerged in polyalkylene glycol and aged in an oven purged with nitrogen. In the current study, a setup of eight experiments was used to understand the effect of three major processing factors on the properties of the silicone rubber. Experiments were designed based on a two-level factorial design of experiment (DOE) with factors of mechanical strain, exposure to heat transfer fluid, and temperature. Samples were aged for six weeks and then characterised in terms of hardness, tear strength, tensile properties, thermal properties, and surface chemistry. Furthermore, DOE analysis was performed to determine the significance of each ageing factor as well as their interaction effects on the silicone rubber’s properties.

## 2. Experimental

### 2.1. Design of Experiment (DOE)

The processing parameters affecting the silicone rubber’s (SR’s) lifetime in the present study are: Temperature, medium, and strain. To analyse the effect of these factors, DOE was employed as a tool of a systematic experimental design, enabling an investigation of the effects of each factor and their interactions on the SR’s properties. Two levels were considered for each factor: At rest condition and at the most severe condition experienced during processing. Experiments were designed based on a balanced 2^3^ full factorial model. The factors and their levels are shown in Table 1. The ageing trials and the allocated sample codes are summarised in Table 2.

### 2.2. Materials

A translucent silicone rubber with a Shore-A hardness of 35, tensile strength of 10.67 MPa, tear strength of 44.9 kN/m, and elongation at break of 1149% (as per the product’s technical data sheet) was used in this study. Polyalkylene glycol (PAG) was used as the heat transfer fluid in the present study with the following properties, as per the product’s technical data sheet: Flash point of 246 °C, fire point of 285 °C, and a pour point of −34 °C, specific gravity of 1.051, and specific heat capacity of 1.95 kJ·kg^−1^K^−1^ at 20 °C.

### 2.3. Accelerated Aging Experiment

**Figure 1:**
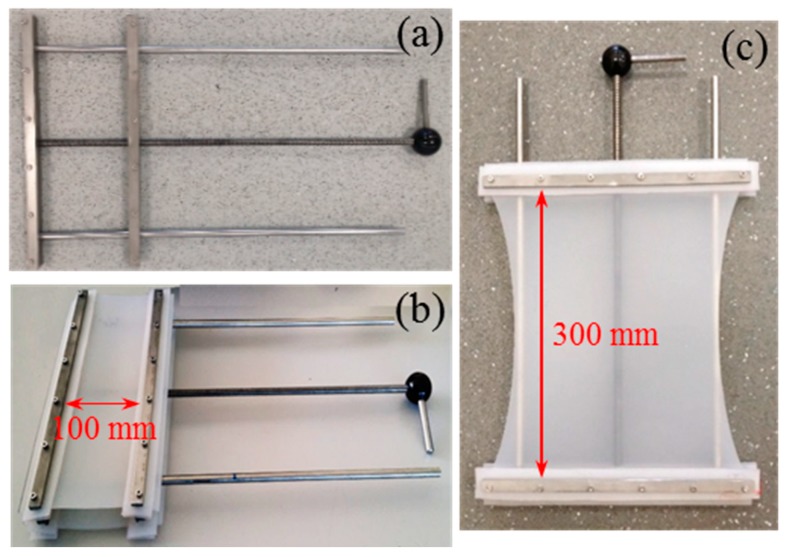
(**a**) Special rig designed for straining SR, (**b**) unstrained SR framed in the rig, (**c**) SR under 200% strain.

The first four ageing tests were set up in a controlled temperature room at 22 °C, while trials 5–8 were set up in a convection oven at 180 °C. To put the SR under strain, a special straining frame was designed, as shown in Figure 1a, and four frames were manufactured in-house. Two oil baths filled with PAG, at 22 and 180 °C, were prepared for trials 3–4 and 7–8, respectively. SR samples were aged for six weeks for all 8 conditions described in Table 2. Once the experiment was finished, the SR samples were removed from the oil baths and unstrained. PAG was removed from the surface of the samples using paper towels and then all 8 samples were dried in a vacuum oven at 30 °C for one week prior to the characterisation.

### 2.4. Characterisation

**Figure 2:**
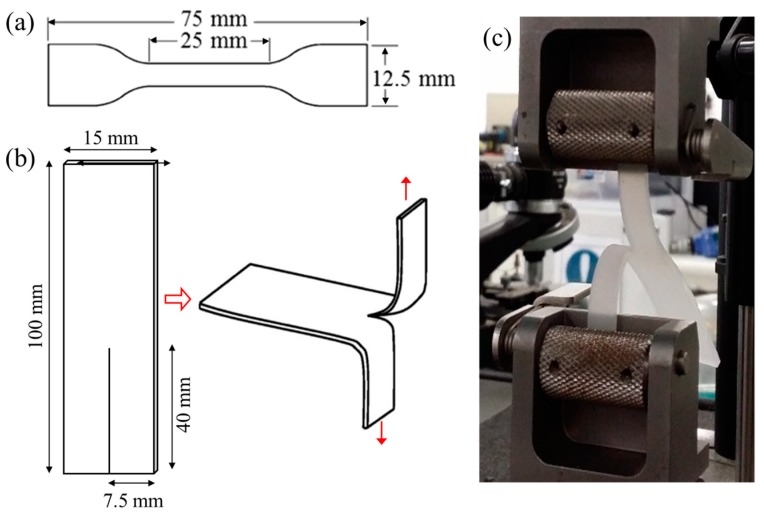
(**a**) Type-2 dumbbell specimen for tensile testing according to AS 1683.11-2001, (**b**) trouser specimen for tear testing according to AS 1683.12-2001, (**c**) self-tightening grips with the trouser specimen in the machine.

The mechanical properties of the SR samples were determined via tensile, tear, and hardness measurements. Tensile tests were performed using an Instron 4467 Universal testing machine with self-tightening roller grips (Figure 2c) according to the AS 1683.11-2001 (ISO 37) standard method. Self-tightening grips are ideal for testing elastomers, preventing specimen slippage. Six type-2 dumbbell-shaped specimens (Figure 2a) were cut out of each SR sample. Measurements were performed at ambient temperature with a 100 N load cell and a crosshead speed of 500 mm/min. Tear tests were conducted on the same instrument and according to the AS 1683.12-2001 (ISO 34) standard method with trouser specimens (Figure 2b). The instrument was operated without interruption at a constant rate of 100 mm/min until the samples teared. Six trouser specimens were tested for each sample. The hardness of the samples was measured by using a Micro-O-Ring hardness tester (Shore Instruments-Instron) according to the ASTM D2240 standard method at room temperature (22 °C). Ten indentation values were recorded for each sample.

The thermal properties of the samples were investigated by differential scanning calorimetry (DSC) [19,20] with a Netzsch 214 Polyma under nitrogen, following a cool-heat-cool-heat cycle according to the following procedure: (1) Holding for 2 min at 20 °C, cooling down to −160 °C at a cooling rate of 10 °C/min, (2) holding at −160 °C for 5 min, heating up to 100 °C at a heating rate of 10 °C/min, (3) holding at 100 °C for 5 min, cooling down to −160 °C at a cooling rate of 10 °C/min, (4) holding at −160 °C for 5 min, heating up to 100 °C at a heating rate of 10 °C/min. Liquid nitrogen was used to achieve the low temperatures for the DSC measurements. To investigate the changes in the chemical composition of the SR with ageing, a Bruker Vertex 70 Fourier transform infrared (FTIR) (Bruker, Germany) spectrometer was used. The data was obtained in the attenuated total reflectance (ATR) mode, using 64 scans at a 4 cm^−1^ resolution from 4000 to 600 cm^−1^.

## 3. Results and Discussion

### 3.1. Hardness

Various applications of elastomers, in particular when used in industrial processes, require the material to perform well in terms of its mechanical properties [2,21]. The hardness, tear strength, tensile strength, elongation at break, and modulus of the silicone rubber are of significant importance for the manufacturing process of the present study. The effects of different ageing conditions on the mechanical properties of the silicone rubber are as follows.

**Figure 3:**
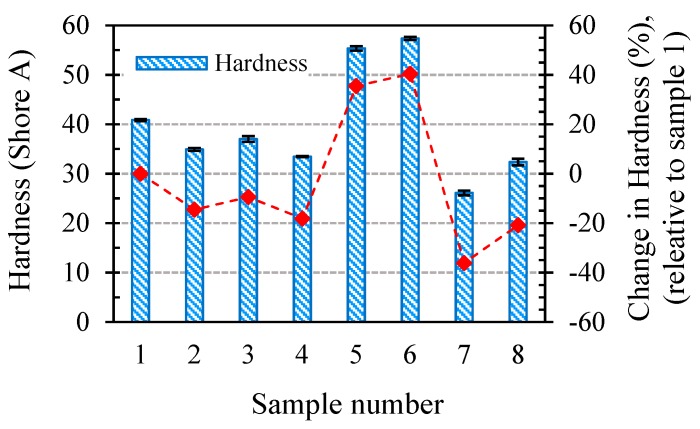
Effect of different ageing conditions on the hardness of the SR.

Figure 3 shows the variations of the SR’s hardness after a six-week exposure to different ageing conditions. The changes in the hardness as a percentage of the unaged SR hardness are reported on the left-hand vertical axis. It is observed that the hardness decreases for all ageing conditions except for samples aged in air at a temperature of 180 °C (samples 5 and 6), which show a significant increase. The highest hardness value belonged to sample 6 (57.35 Shore A), exhibiting a 40% increase compared to the unaged SR (sample 1) with a hardness of 41 Shore A. The hardness of sample 7, aged in PAG at 180 °C, exhibited a decrease of 37%, reaching its lowest value of 26.1 Shore A. An interesting observation from Figure 3 is that straining the SR can both decrease and increase the hardness depending on the temperature. Comparing sample 2 with sample 1, and sample 4 with sample 3, it is seen that strained samples aged at a low temperature of 22 °C showed a lower hardness compared to their counterparts. On the other hand, strained samples at a high temperature have higher hardness values compared to not-strained samples, irrelevant of the aging medium, as evident by comparing sample 6 with sample 5 and sample 8 with sample 7.

### 3.2. Tear Testing

**Figure 4:**
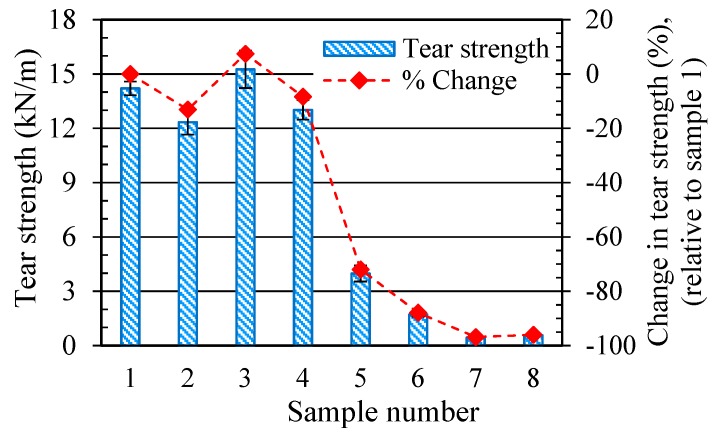
Variations of the tear strength of SR with ageing conditions.

The effect of the ageing conditions on the SR’s tear strength is illustrated in Figure 4. It is observed that tear strength decreases drastically when the SR is exposed to high temperatures. The tear strength of unaged SR (A22-0%) is 14.2 kN/m, which drops to only 3.9 kN/m in sample 5 (A180-0%) upon ageing at 180 °C, exhibiting a 72% reduction. When aged in PAG at 180 °C, the tear strength decreases to about 95% of that of unaged SR. In contrast to samples aged at high temperatures, exposure to PAG at a low temperature of 22 °C slightly enhances the tear strength; sample 3 (P22-0%) has a tear strength of 15.7 kN/m, which is 7.4% higher than that of unaged SR (A22-0%). The tear strength of sample 4 (P22-200%), with a value of 13.01 kN/m, is also slightly higher than that of sample 2 (A22-200%). This may be due to the PAG residue in samples 3 and 4. On the other hand, samples aged at high temperature PAG have a significantly lower tear strength compared to those aged in high temperature air. This could be attributed to the adverse effect of the attack of by-products of PAG thermal degradation on the rubber. FTIR analysis of PAG showed that it became acidic during the ageing period. As seen in Figure 7b (in Section 3.4), FTIR spectra reveals the appearance of a peak at a wavenumber of 1725 cm^−1^ with an increased height as PAG is aged. This peak is indicative of the presence of a C=O [22] bond and its increased intensity with thermal ageing suggests the intensified acidity in PAG.

**Figure 5:**
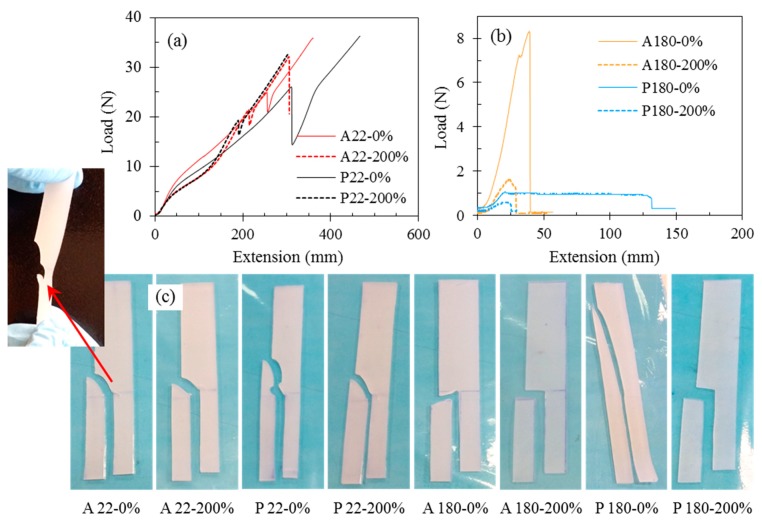
Tear profiles of SR specimens aged at (**a**) 22 °C and (**b**) 180 °C. (**c**) Illustration of teared specimens. It should be noted that in this figure only one specimen is shown for each sample for clarity of the graphs.

The SR teared specimens and their tear profiles are depicted in Figure 5. It can clearly be observed that the tear behaviour of the SR changes with ageing. The first four samples, including unaged SR (sample 1), exhibit a tear behaviour with several peaks (Figure 5a) while SR samples aged in the oven show one small peak (Figure 5b) with a smooth constant tearing. The teared specimens (Figure 5c) also illustrate this difference.

### 3.3. Tensile Testing

**Figure 6:**
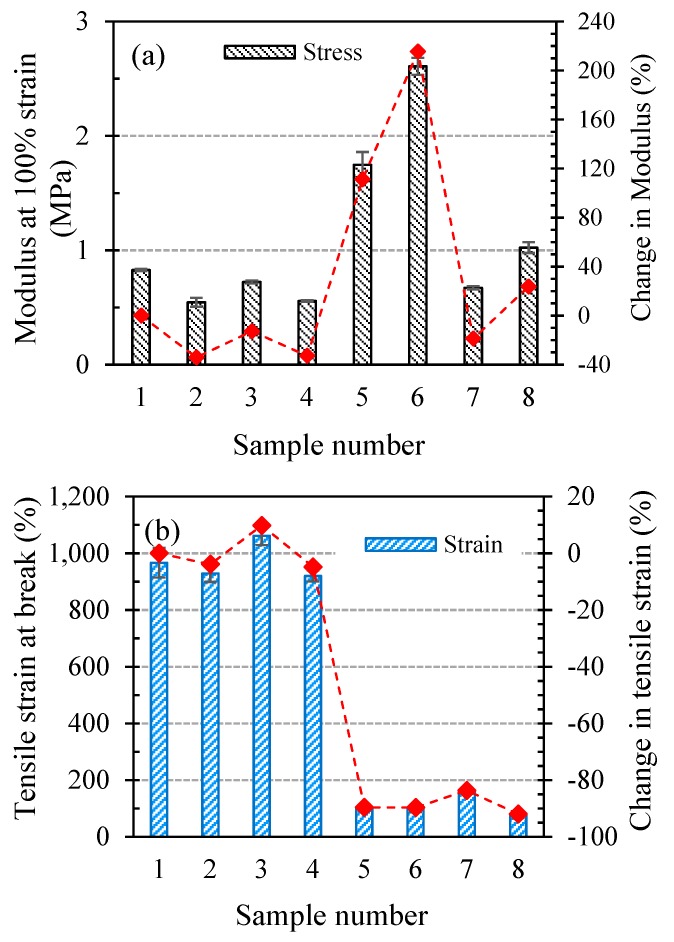

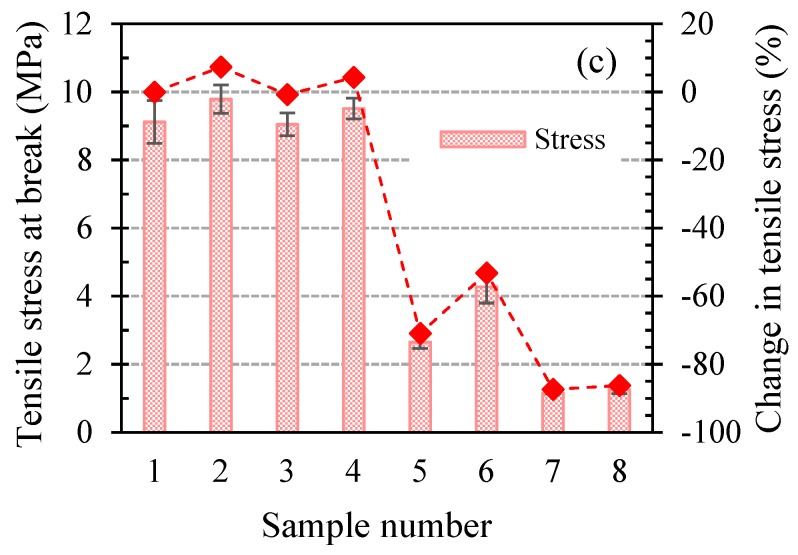
Tensile properties of SR samples, (**a**) modulus at 100% strain, (**b**) tensile strain at break, and (**c**) tensile stress at break.

The tensile properties of SR, in particular, the modulus and elongation at break, are of significant importance for the industrial application which is being studied in the present article. Figure 6 depicts the effect of ageing conditions on the tensile properties of SR. The modulus at 100% strain is reported for the SR samples instead of the Young’s modulus, a common practice for elastomeric materials. The unaged SR has a modulus of about 0.83 MPa. Samples 2, 3, and 4, which were aged at 22 °C, show lower modulus values than that of unaged SR. On the other hand, the moduli of samples aged at 180 °C are higher. Samples 5 and 6 with moduli of 1.747 and 2.608 MPa demonstrate very high increments of 111% and 215% compared to unaged SR, respectively. Similar to the hardness behaviour of samples in Figure 3, strained samples at 22 °C have lower moduli compared to their unstrained counterparts, while samples 6 and 8 exhibit significantly higher moduli compared to samples 5 and 7, respectively.

The tensile strain at break or elongation at break of the SR samples in Figure 6b also illustrates variations comparable to those of the tear strength under the effect of different ageing environments. The elongations at break of the samples aged at 180 °C are markedly lower than those of samples aged at 22 °C. Furthermore, samples 2 and 4 (strained at 22 °C) show elongations slightly lower than samples 1 and 3, respectively, resembling their tear behaviour. Figure 6c reveals that exposure to PAG at low temperatures does not affect the tensile strength of the SR while straining the SR slightly enhances its tensile strength. On the other hand, when comparing samples 5 and 6 with samples 7 and 8, it is obvious that high temperature PAG decreases the SR tensile strength far more severely than high temperature air.

### 3.4. FTIR

**Figure 7:**
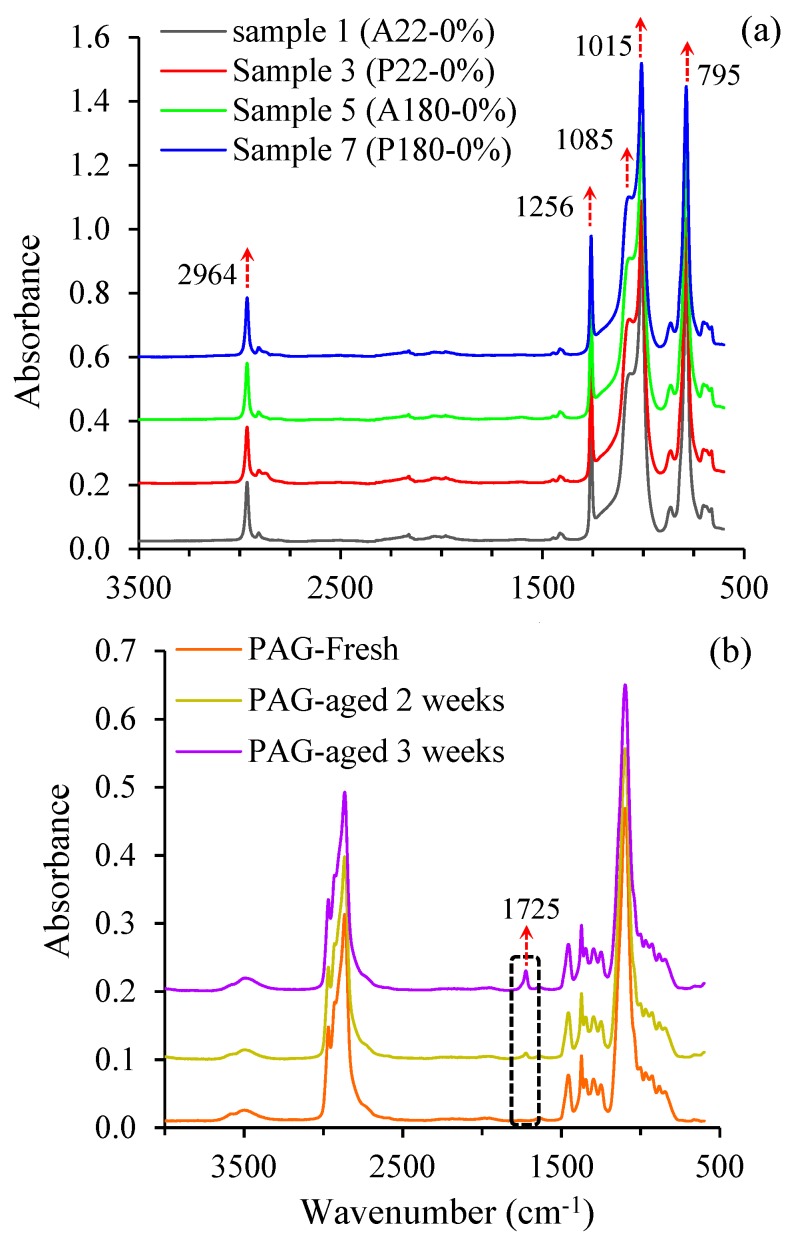
FTIR spectra of (**a**) SR samples and (**b**) PAG samples.

To determine whether exposure to PAG and/or high temperatures affect the chemical structure of SR, attenuated total reflectance-FTIR analysis was conducted on all SR samples. The FTIR spectra of samples 1, 3, 5, and 7 are shown in Figure 7a. The five characteristic peaks for silicone rubber are observed at 2964, 1256, 1015–1085, and 795 cm^−1^, which correspond to the vibrations of methyl (–CH_3_), Si–CH_3_ bond, Si–O–Si, and Si–(CH_3_)_2_, respectively [12,23]. As evident from Figure 7a, there is no detectable changes in the FTIR spectra of aged samples of 3, 5, and 7 compared with the FTIR spectra of unaged sample 1. This reveals that the surface chemistry of the SR is not impacted by either of the ageing environments. It is noteworthy to mention that the FTIR spectra of samples 2, 4, 6, and 8, which are not shown here for clarity of the graph, were similar to the ones illustrated in Figure 7a. Considering that straining is a mechanical phenomenon, such an observation is expected. In a previous study on SR’s ageing behaviour, a similar stability in its chemical structure was observed [2], which was attributed to the non-existence of oxidative agents due to purging the oven with nitrogen. However, it appears that even the presence of oxygen in the oven at a high temperature of 180 °C as in the present study does not change the surface chemistry of SR.

### 3.5. Thermal Properties

**Figure 8:**
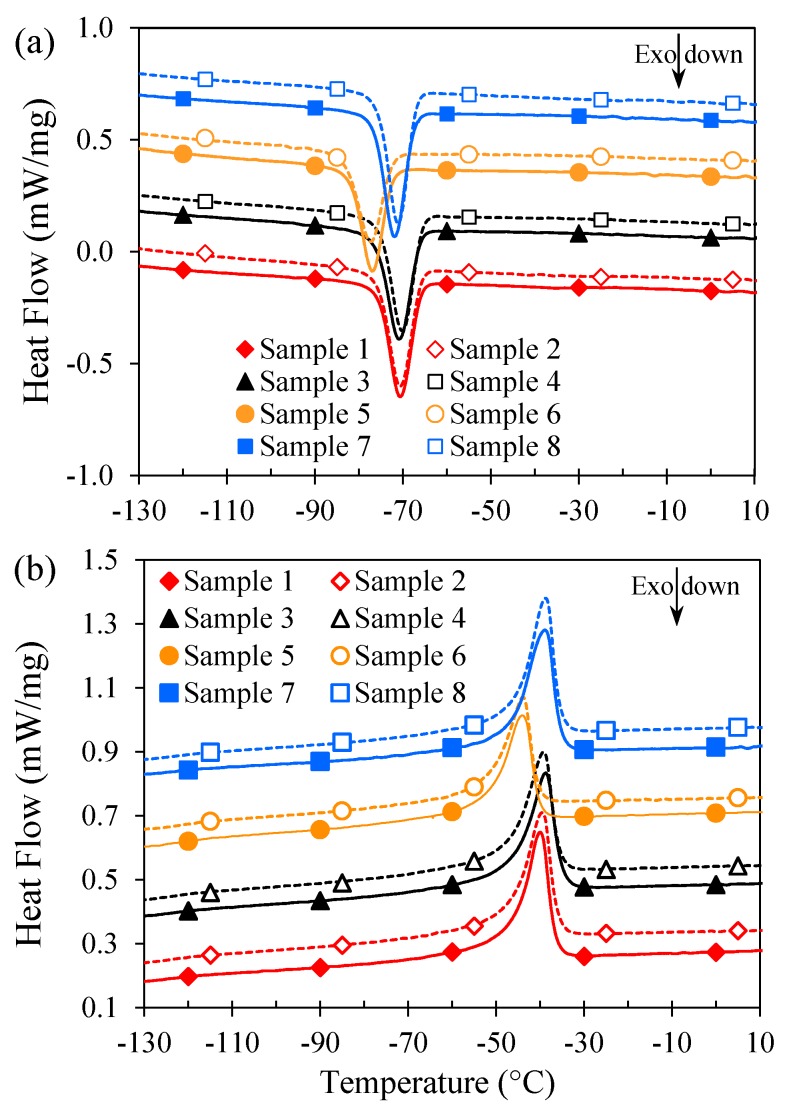
Comparative DSC thermograms of SR samples in the (**a**) second cooling cycle and (**b**) second heating cycle.

**Figure 9:**
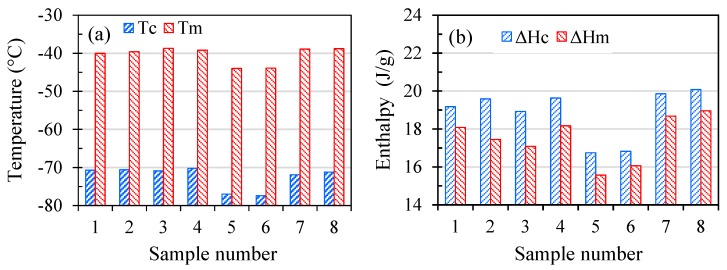
Variations of thermal properties of SR samples, (**a**) temperatures of crystallisation, *T*_c_, and melting, *T*_m_. (**b**) Enthalpies of crystallisation, Δ*H*_c_, and melting, Δ*H*_m_.

While FTIR analyses did not demonstrate any variations among differently aged SR samples, DSC measurements showed that samples that were aged in air in the oven had significantly different DSC thermograms during both cooling and heating cycles as illustrated in Figure 8. The variations of the crystallisation temperature (*T*_c_) and crystallisation enthalpy (Δ*H*_c_) obtained from the cooling cycles and the melting temperature (*T*_m_) and melting enthalpy (Δ*H*_m_) obtained from the heating cycles are shown in Figure 9 for all SR samples.

Unaged SR has a *T*_c_ and *T*_m_ of about −40 and −70.7 °C, respectively. All aged samples are observed to have similar values of *T*_c_ and *T*_m_ except those aged at high temperatures in air. Samples 5 and 6 with a *T*_c_ and *T*_m_ of about −44 and −77 °C, respectively, exhibit a decrease of about 10% in their *T*_c_ and *T*_m_ values. In a comparable manner, while the Δ*H*_c_ and Δ*H*_m_ of samples 2, 3, 4, 7, and 8 have values close to those of the Δ*H*_c_ and Δ*H*_m_ of unaged SR, samples 5 and 6 show a 12–14% reduction. This suggests that samples 5 and 6 have smaller degrees of crystallinity compared to other samples. It is important to note that the DSC profiles and data reported in Figure 8 and Figure 9 are from the second cooling and heating cycles and therefore the discrepancies could not be due to the thermal history of the samples since the first heating cycles have erased the thermal history of the samples [20,24]. It is also worth mentioning here that the second cooling and heating cycles for all samples superimposed the first cooling and heating cycles almost completely. The difference in the thermal properties of samples 5 and 6 might be due to different degrees of crosslinking compared to other samples. However, further investigation is required to confirm this speculation.

### 3.6. DOE Analysis: Main and Interaction Effects

**Figure 10:**
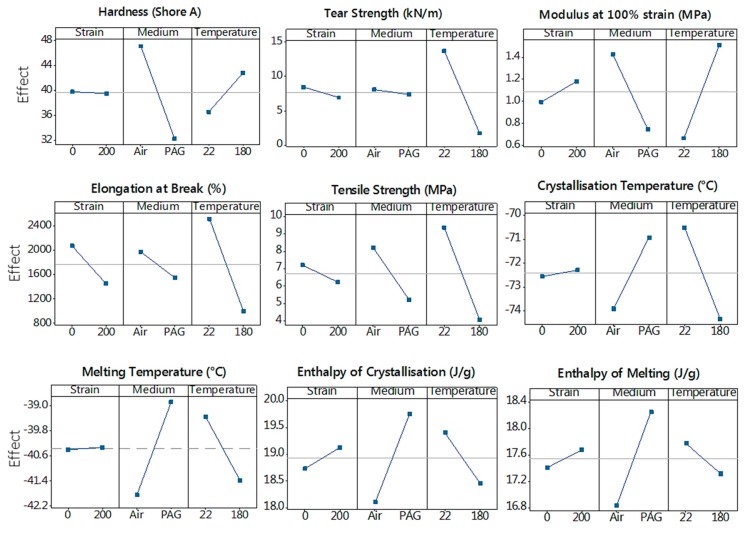
Main factors effects on the properties of the SR samples.

Data obtained on the mechanical and thermal properties of SR samples from the ageing experiment was analysed in Minitab® 17.1.0 for the two-level full factorial DOE presented in Table 2. The main effects of the ageing factors on the SR’s different properties are reported in Figure 10. The effect for each factor is determined by averaging the response values at the high level (+) and low level (−) for that factor. Table 3 presents an example for factor A in a two-level, three-factor (2^3^) factorial design and how the main effect is calculated for this factor. Figure 10 shows that the medium and temperature are the two most determining factors on SR ageing while strain seems to have the least impact and in some cases, even a negligible effect, such as for hardness. It is noteworthy to mention that the mechanical strain put on the SR sample was a constant strain and it is expected that the SR would behave differently under cyclic loading. For the purpose of the present study, constant strain was chosen rather than cyclic. In Figure 10, the steeper the line for a factor, the stronger its effect on the response.

**Figure 11:**
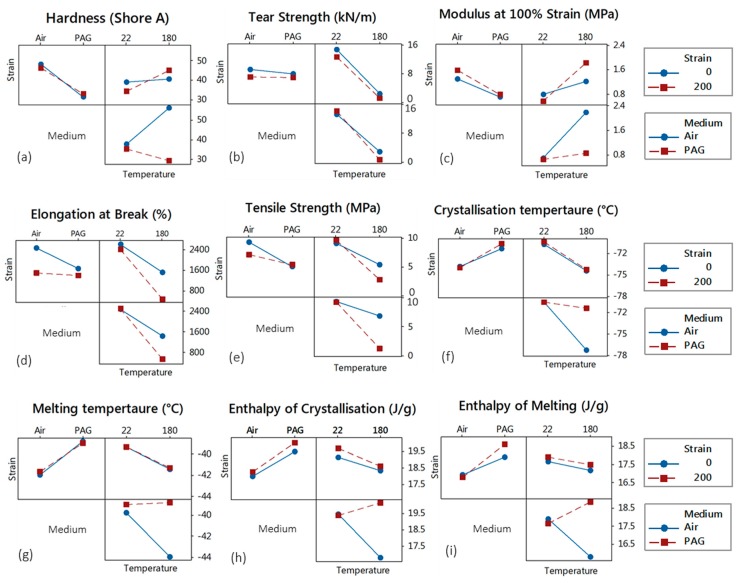
Interaction effects of ageing factors on the mechanical and thermal properties of the SR samples.

The contrasts of averages determined in the factorial design provide statistical power to the estimated effects compared to the one-factor-at-a-time (OFAT) experiments, which require more runs to provide the equivalent precision power. In addition to the reduced number of runs, revealing the impacts of the “interactions” of factors on the response is another significant aspect of DOE [7]. It is important to investigate the interactions since if their effects on the response are significant, one cannot interpret the main effects without considering the interaction effects [8,25]. Figure 11 depicts the two-way interaction plots for the strain, medium, and temperature of the SR samples. In these plots, the parallel and non-parallel lines are indicative of the non-existence and existence of an interaction between each two factors, respectively. The more non-parallel the lines are, the greater the strength of the interaction. Figure 11 shows that there are some interactions between the ageing factors. A strong interaction between the medium and temperature is observed for the hardness, followed by the temperature/strain interaction and a negligible strain/medium interaction (Figure 11a). For the tear strength, the plots reveal negligible to no interactions (Figure 11b). For the tensile properties, however, interactions are observed between all the ageing factors (Figure 11c–e). On the other hand, the plots for the thermal properties show a very strong interaction between medium and temperature with negligible to no interactions among other factors.

**Figure 12:**
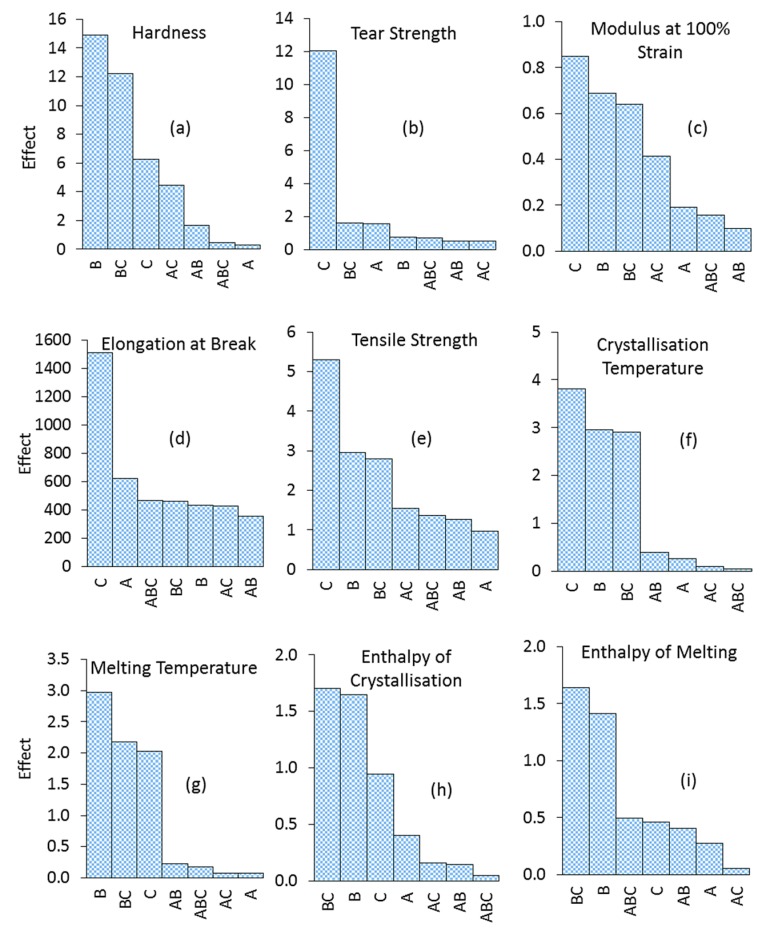
Pareto charts illustrating the effects of ageing factors (A: strain, B: medium, C: temperature) and their two- and three-way interactions (AB, AC, BC, ABC) on the SR properties.

Factors’ interactions effects can be more clearly observed from the Pareto charts. Figure 12 illustrates the Pareto charts for the experimental factors of the present study. These charts depict the relevant significance of the ageing factors’ main and interaction effects on the mechanical and thermal properties of SR. As was seen in Figure 11a, Figure 12a also shows that the medium/temperature interaction (BC) and strain/temperature interaction (AC) are much more significant than the strain/medium (AB) and the three-way interaction (ABC). It is also interesting to note that the medium/temperature interaction has a larger impact on the hardness than the temperature. In accordance with Figure 11b, Figure 12b clearly illustrates the very small effects of the factors’ interactions on the SR’s tear strength. It is observed that the effect of temperature is far more significant than those of the other ageing factors or the interactions. Temperature is also found to be the most influential factor on the SR’s tensile properties. However, as seen in Figure 12c,d, the medium and strain as well as the factors’ interactions affect the tensile strength, elongations, and modulus of the SR. On the other hand, the thermal properties of SR are mostly influenced by the temperature, medium, and their two-way interaction (Figure 12f–i).

## 4. Conclusions

The design of the experiment was used to investigate the effects of three ageing factors and investigate their interactions on the physical properties of silicone rubber used in a particular industrial process. Statistical analysis revealed that temperature and medium were the most influential ageing factors. The temperature/medium interaction was also observed to have a significant effect on the SR’s properties. The mechanical properties of SR were found to be more susceptible to deterioration than the thermal properties. The hardness decreased for all ageing conditions expect for samples aged in high temperature air and it reached a value of 57.35 in sample A180-200%, showing an increase of 40%. The tear strength, however, decreased markedly when exposed to both high-temperature air and PAG, with a decrease of 96% in sample P180–200%. For both hardness and tear strength, strained samples showed lower values compared to their unstrained counterparts.

Ageing at 22 °C did not impact the tensile properties of SR samples significantly. In contrast, ageing at 180 °C resulted in a 90% reduction in the elongation at break as well as an 86% reduction in the tensile strength of sample P180-200%. Statistical analysis showed that temperature, medium, and their interaction affected the tensile properties. Strain also showed an impact on the elongation at break of the samples. No appreciable change was detected in the FTIR spectra of the aged samples, suggesting that the SR ageing mechanism is mainly physical. Thermal properties (*T*_c_, *T*_m_, Δ*H*_c_, and Δ*H*_m_) of the SR, determined via DSC measurements, did not demonstrate any significant variation compared to those of unaged SR except for samples aged in air at 180 °C, which showed reduced values.

## Figures and Tables

**Table 1:** Processing factors and their levels used in the DOE approach.

Factor	Low Level	High Level
Temperature	22 °C	180 °C
Medium	Air	PAG
Strain	0%	200%

**Table 2:** The 2^3^ full factorial experimental layout and the sample codes.

Trial	Strain (%)	Medium	Temperature (°C)	Sample Code
1	0	Air	22	A22-0%
2	200	Air	22	A22-200%
3	0	PAG	22	P22-0%
4	200	PAG	22	P22-200%
5	0	Air	180	A180-0%
6	200	Air	180	A180-200%
7	0	PAG	180	P180-0%
8	200	PAG	180	P180-200%

**Table 3:** Example of the calculation of the main effect for one factor in a two-level, three-factor (2^3^) factorial design.

Run	A	Response	Run	A	Response
1	-	$Y_{1}$	5	-	$Y_{5}$
2	+	$Y_{2}$	6	+	$Y_{6}$
3	-	$Y_{3}$	7	-	$Y_{7}$
4	+	$Y_{4}$	8	+	$Y_{8}$

Main effect for factor A: Contrast between the high and the low averages obtained from formulas: $High\;average=\frac{Y_{2}+Y_{4}+Y_{6}+Y_{8}}{4}$; $Low\;average=\frac{Y_{1}+Y_{3}+Y_{5}+Y_{7}}{4}$.
